# A Stability-indicating HPLC Method for Assay of Lercanidipine Hydrochloride in Tablets and for Determining Content Uniformity

**DOI:** 10.4103/0250-474X.70490

**Published:** 2010

**Authors:** H. O. Kaila, M. A. Ambasana, R. S. Thakkar, H. T. Saravaia, A. K. Shah

**Affiliations:** National Facility for Drug Discovery through New Chemical Entities Development and Instrumentation support to Small Manufacturing Pharma Enterprises, Department of Chemistry, Saurashtra University, Rajkot - 360 005, India

**Keywords:** Calcium channel blocker, column liquid chromatography, degradation, stability- indicating, lercanidipine hydrochloride

## Abstract

A simple, precise and accurate HPLC method has been developed and validated for assay of lercanidipine hydrochloride in tablets and for determination of content uniformity. An isocratic separation was achieved using a Chromasil YMC Pack C_8_, 150 × 4.6 mm i.d., 5µm particle size columns with a flow rate of 1 ml/min and using a UV detector to monitor the elute at 240 nm. The mobile phase consisted of 0.02 M ammonium dihydrogen phosphate buffer:methanol (35:65, v/v) with pH 3.5 adjusted with phosphoric acid. The method was validated for specificity, linearity, pre-cision, accuracy, robustness and solution stability. The specificity of the method was deter-mined by assessing interference from the placebo and by stress testing of the drug (forced degradation). The method was linear over the concentration range of 20-80 µg/ml (r^2^= 0.9992) with a limit of detection and quantitation of 0.1 and 0.3 µg/ml respectively. Intraday and interday system and method precision were determined and accuracy was between 99.3-101.9 %. The method was found to be robust and suitable for assay of lercanidipine hydrochloride in a tablet formulation and for determination of content uniformity. Degradation products resulting from the stress studies did not interfere with the detection of lercanidipine hydrochloride and the assay is thus stability-indicating.

Lercanidipine hydrochloride, a calcium-channel blocker, which is chemically 2[(3,3-diphenylpropyl)methylamino]-1,1-dimethylethylmethyl-2,6-dimethyl-4-(3-nitrophenyl)-1,4-dihydropyridine-3,5-dicarboxylate (fig. 1). Its molecular formula is C_36_H_41_N_3_O_6_HCl and molecular weight 648.19. Lercan-idipine is used for treating angina pectoris and hypertension[1].

**Fig. 1:**
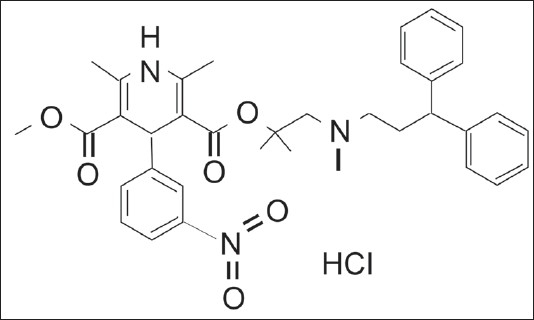
Structure formula of lercanidipine hydrochloride

There are few references to the analysis of lercanidipine hydrochloride and its impurities in pharmaceutical dosage forms[2] and UV spectrophotometric determination of lercanidipine hydrochloride in bulk and tablet are reported[3]. The development and validation of high performance liquid chromatographic method for estimation of lercanidipine in rabbit serum[4] and determination of lercanidipine and its impurities using DryLab software also reported[5]. Only one HPLC method reported in the literature for the assay determination of lercanidipine hydrochloride in bulk and pharmaceutical dosage form[6]. There are no reports of methods for study of the effect of stress on pharmaceutical dosage forms and there is no validated HPLC method, which enable both assay and determination of content uniformity of lercanidipine hydrochloride in pharmaceuticals dosage forms.

The objective of this work was to de-velop a stability-indicating liquid chromatographic analytical method for assay of lercanidipine hydrochloride and for determination of the content uniformity of a tablet formula-tion. The validation procedure followed the guidelines of ICH[7] and USP 30[8].

Lercanidipine hydrochloride reference standard was provided by Glenmark Pharmaceuticals, Ankleshwar, India. Tablets of lercanidipine hydrochloride, Lerka (10 mg, Piramal HealthCare, Mumbai, India) were procured from a local pharmacy. All chemicals used were of analytical grade. Methanol and water both HPLC grade, were from spectrochem (Mumbai, India). Nylon syringe filters 0.45 µm were from Millex-HN (Mumbai, India).

Liquid chromatography was performed with Waters equipment with TM 600 quaternary pump, Waters 2489 UV/Vis detector, Waters 600 controller, Waters in-line degasser AF and manual injector with 20 µl loop. The equipment was connected to a multi-instrument data-acquisition and data-processing system (Empower software). The chromatographic separation was performed using a Chromasil YMC Pack C_8_, 5 µm, 150×4.6 mm i.d. column. Separation was achieved using a mobile phase consisted of 0.02 M ammonium dihydrogen phosphate buffer pH 3.5-methanol (35:65, v/v) solution at a flow rate of 1 ml/min. The eluent was monitored using UV detection at a wavelength of 240 nm. The column was maintained at 30° temperature and injec-tion volume 20 µl was used. The total runtime was 10 min. The mobile phase was filtered through 0.45 µm micron filter prior to use.

A stock solution (500 µg/ml) of lercanidipine hydrochloride reference standard was prepared in water:methanol (30:70, v/v). To prepare standard solution 50 µg/ml for assay 5 ml standard stock solution was transferred to 50 ml volumetric flask and volume was adjusted with water:methanol (30:70, v/v). To prepare a stock solution (500 µg/ml) for assay, 20 tablets were weighed and mixed. An aliquot of powder equivalent to the weight of 5 tablets was accurately weighed and transferred to 100 ml volumetric flask and dissolved in 40 ml of water:methanol (30:70, v/v) and the mixture was sonicated for 30 min. The contents of the flask were then left to return to room temperature and volume was adjusted with the same solvent mixture. This solution 10 ml was filtered through a 0.45 µm nylon syringe filter. To prepare test solution 50 µg/ml for assay 5 ml test stock solution was transferred to 50 ml volumetric flask and volume was adjusted with water:methanol (30:70, v/v). For content uniformity one tablet was taken to prepare test solution 50 µg/ml.

To perform the forced degradation study drug was subjected to acidic, alkaline, oxidizing, thermal and photolytic conditions[9]. For acidic deg-radation the drug was heated under reflux with 1 N HCl at 80° for 1 h and the mixture was neutralized. For alkaline degradation the drug was treated with 0.1 N NaOH at room temperature for 100 min and the mixture was neutralized. For degradation under oxidizing conditions the drug was heated under reflux with 3% hydrogen peroxide at 80° for 1 h. For thermal degrada-tion the powdered drug was exposed at 70° for 72 h. For photolytic degra-dation the powdered drug was exposed to sunlight for 72 h. The placebo was also subjected to the same stress conditions to determine whether any peaks arose from the declared excipients. After completion of the treatments the solutions were left to return to room temperature and diluted with water:methanol (30:70, v/v) to furnish 50 µg/ml solutions. fig. 2 shows the chromatogram of untreated drugs in tablet solution.

**Fig. 2:**
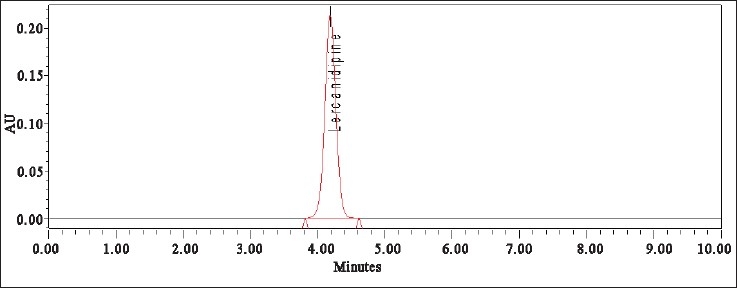
Chromatogram of untreated drug in tablet solution

In this work an analytical HPLC method for assay and determination of content uniformity of lercanidipine hydrochloride in a tablet formulation was developed and validated. The basic chromatographic conditions were designed to be simple and easy to use and reproduce and were selected after testing the different conditions that affect HPLC analysis, for example column, aqueous and organic components of the mobile phase, proportion of mobile phase components, detection wavelength, diluents, concentration of analyte etc. The Chromasil YMC Pack C8 column was used because of its advantages of high resolving capacity, better reproducibility, low-back pressure and low tailing. For mobile phase selection, preliminary trials using mobile phases of different composition containing water adjusted to acid pH by addition of orthophosphoric acid and methanol resulted in poor peak shape. When water was replaced by ammonium dihydrogen phosphate buffer adjusted to acid pH by addition of orthophosphoric acid better peak shape was obtained. The proportion of the mobile phase components was optimized to reduce retention times and enable good resolution of lercanidipine hydrochloride from the degradation products. A detection wavelength of 240 nm was selected after scanning the standard solution over the range 190-370 nm by use of the UV spectrophotometer. Detection at 240 nm resulted in good response and good linearity.

The specificity of the method was determined by checking for interference with the drug from placebo components. In forced degradation study, major degradation 44% occurred under alkaline conditions. Under acidic conditions the drug was degraded by approxi-mately 7%. The drug was approximately 15% degraded under oxi-dizing conditions. The drug was degraded 6% under thermal condition and 10% degradation occurred under photolytic conditions.

To determine linearity a calibration graph was obtained by plotting lercanidipine hydrochloride concentration against peak area. Linearity was good in the concentration range 20-80 µg/ml. The regression equation was *y*=48,443*x*+20,928 where *x* is the concentration in µg/ml and *y* is the peak area in absorbance units; the correlation coefficient was 0.9992.

For assay (n=6) and determination of content uniformity (n=10), RSD for the system precision was 0.43% and 0.37%, respectively, on the same day (intraday) and 0.40% and 0.55% on different days (interday). The mean values of method precision (repeatability) were 101.5%, RSD 0.28% for assay and 101.4%, RSD 0.46% for content uniformity on the same day (intraday) and 100.8%, RSD 0.61% for assay and 100.9%, RSD 0.82% for content uniformity on different days (interday). Intermediate precision was established by determining the overall (intraday and interday) method precision for assay and determination of content uniformity. For intermediate precision, overall assay value (n=12) was 101.2%, RSD 0.37% and overall content uniformity (n=20) was 101.4%, RSD 0.70%. The precise result for content uniformity was indicative of uniform distribution of the drug in the tablets without significant variation; this is in accordance with the USP[8], which stipulates acceptance limits for drug content uniformity and RSD as 85-115 and <6%, respectively.

The accuracy of the method was assessed by determination of recovery for three concentrations (corresponding to 50, 100 and 150% of test solution concentration) covering the range of the method. For each concentration three sets were prepared and injected in duplicate. The mean recovery of lercanidipine hydrochloride was between 99.3-101.9% and RSD of recoveries between 0.4-1.44 %.

The robustness of the method was evalu-ated by assaying test solutions after slight but deliberate changes in the analytical conditions flow rate (±0.1 ml/min), the proportions of buffer:methanol (33:67 and 37:63, v/v) and changing the column temperature 35°. For each different analytical condition the standard solution and test solution were prepared separately. The result obtained from assay of the test solution was not affected by varying the conditions and was in accordance with the true value. System suitability data were also found to be satisfactory during variation of the analytical conditions. The analytical method there-fore remained unaffected by slight but deliberate changes in the analytical conditions.

Stability in solution was evaluated for the standard solution and the test preparation. The solutions were stored at 5° and at ambient temperature. Without protection of light and tested after 12, 24, 36 and 48 h. the responses for the aged solution were evaluated by comparison with freshly prepared solutions. During study of the stability of stored solutions of standards and test prepara-tions for assay determination the solutions were found to be stable for up to 36 h[10]. Assay values obtained after 36 h were statistically identical with the initial value without measurable loss.

Before each measurement of valida-tion data a system suitability test was performed by measurement of general characteristics such as peak asymmetry, number of theoretical plates and % RSD of peak area observed for a standard solution. The values obtained were satisfactory and in accordance with in-house limits (Table 1).

**TABLE 1:** SYSTEM SUITABILITY DATA

System suitability data In-house limit	% RSD^a^ NMT^b^ 2.0	Theoretical plates NLT^c^ 3000	Asymmetry NMT^b^ 2.0
Validation data			
Specificity	0.52	4221	1.02
Linearity	0.43	4324	1.00
Limit of detection	0.34	4421	1.03
Limit of quantitation	0.41	4213	1.01
Method precision			
For assay	0.43	4532	1.00
For content uniformity	0.37	4357	0.98
Intermediate precision			
For assay	0.40	4231	1.01
For content uniformity	0.55	4436	0.99
Accuracy	0.34	4315	1.00
Solution stability	0.27	4231	1.01
Robustness	0.46	4328	1.02

a relative standard deviation

b not more than

c not less than
